# Mentalizing the therapist – Therapist experiences with short-term mentalization-based therapy for borderline personality disorder: A qualitative study

**DOI:** 10.3389/fpsyt.2023.1088865

**Published:** 2023-03-16

**Authors:** Amanda Ark Søndergaard, Sophie Juul, Stig Poulsen, Sebastian Simonsen

**Affiliations:** ^1^Stolpegaard Psychotherapy Centre, Mental Health Services in the Capital Region of Denmark, Gentofte, Denmark; ^2^Copenhagen Trial Unit, Department of Regional Health Research, Faculty of Health Sciences, University of Southern Denmark, Copenhagen, Denmark; ^3^Department of Psychology, Faculty of Social Sciences, University of Copenhagen, Copenhagen, Capital Region of Denmark, Denmark

**Keywords:** short-term psychotherapy, mentalization-based therapy, borderline personality disorder, therapist experiences, treatment termination, thematic analysis

## Abstract

**Background:**

Mentalization-Based Therapy (MBT) was originally developed as a structured psychotherapy approach developed to treat borderline personality disorder (BPD) lasting up to 18 months in outpatient settings. However, a short-term (5 months) MBT program has recently been developed. No studies have investigated how MBT therapists experience the shift towards conducting short-term MBT for BPD.

**Objective:**

The objective of this study was to explore therapist experiences with conducting short-term MBT for outpatients with BPD in the Danish mental health services.

**Methods:**

Semi-structured qualitative interviews were conducted with seven therapists about their experiences with short-term MBT after a one-year pilot phase. The interviews were verbatim transcribed and analyzed using thematic analysis.

**Results:**

The following four major themes from the therapists’ experiences with short-term MBT were found in the qualitative analysis: (1) *The longer the better*, (2) *Change processes can be intellectual or experiential*, (3) *Short-term therapy is hard work*, and (4) *Termination is more challenging in short-term MBT*.

**Conclusion:**

Most therapists were overall reluctant towards changing from long-term to short-term MBT. These therapist experiences could inform implementation of short-term MBT in mental health settings in the future.

## Introduction

Psychotherapy programs currently offered for patients with borderline personality disorder (BPD) are often lengthy and resource intensive (1, 2). However, a recent Cochrane review investigating the efficacy of psychotherapies for BPD explored possible differential effects of short versus long psychotherapy in a subgroup analysis and did not find any association between treatment intensity and outcome (3). Since the results from this subgroup analysis are only indirect and should be interpreted with caution, the optimal treatment duration for patients with BPD still remains unclear (4). The length of the current psychotherapies available for BPD creates a barrier to their adoption in the current climate of rising health care costs (2), and outpatient clinics may see a need to implement short-term versions of the treatments usually offered.

Mentalization-based therapy (MBT) is an evidence-supported psychotherapy program for BPD, which was originally manualized as an 18-months program (5). MBT in this format has been shown to reduce self-harm, suicidality, and depression (3). However, a short-term MBT program for BPD has been implemented at the Outpatient Clinic for Personality Disorders at Stolpegaard Psychotherapy Centre in Gentofte, Denmark in a collaboration between the clinic and the research unit at the centre. Recently, a randomised clinical trial named The Short-Term MBT Project has been initiated comparing the effects of short-term (5 months) with long-term (14 months) MBT for outpatients with subthreshold or diagnosed borderline personality disorder (4).

### Mentalization-based therapy for borderline personality disorder

MBT is a psychodynamic psychotherapy, rooted in cognitive theory and attachment theory (5). It was developed specifically for patients with BPD and has shown to be effective compared with treatment as usual (3). Mentalization refers to the capacity to understand one’s own and others’ internal mental states. Patients with BPD are more vulnerable to lose their mentalizing capacity when experiencing emotional distress. The MBT manual offers therapeutic techniques to help bring the patient back into a mentalizing mode (6, 7). However, information about the processes that produce a change in MBT, or in psychotherapy in general, is still limited (8–11).

Even though MBT was originally manualized as long-term program, different durations of MBT are currently offered in outpatient settings around the world (6). In our experience, the idea that longer treatment durations are universally preferable for patients, especially those with more severe psychopathology (12), is pervasive among many therapists practicing MBT. However, the opposite perspective that long-term psychotherapy could be too overwhelming for patients with severe psychopathology, particularly for those with attachment insecurities, could also be prevalent among therapists. To our knowledge, these therapist attitudes have not yet been systematically explored.

### The therapist perspective

Adapting to a short-term version of an existing treatment may be a challenging process for therapists. Yet, no previous empirical research has focused on exploring therapist experiences with delivering short-term MBT, nor are we aware of any qualitative studies focusing directly on the influence of treatment duration on the therapist experience of delivering other types of short-term psychotherapy for BPD patients.

Patients with BPD are often highlighted as a patient group evoking strong emotional reactions in mental health professionals (13–17). Bateman and Fonagy (18) argue that BPD patients are the most difficult patients to treat due to their predominantly ambivalent attachment styles. As a result, therapeutic interventions with this group are often emotionally demanding for the therapist (19). Studies by Betan et al. (20) and Colli et al. (14) on the types of emotional responses evoked by patients with personality disorders found that patients with BPD often elicit strong therapist responses such as feeling helpless, inadequate, overwhelmed, and overinvolved. Reducing the length of the treatment may exacerbate or complicate the emotional responses of the therapist in a way which could potentially influence treatment outcome. Therapist expectancy of treatment outcome is less researched compared to patient expectancy (21, 22). However, these phenomena may be interrelated; e.g. a therapist with a particular response expectancy may consciously or unconsciously communicate this to the patient during treatment, which may ultimately result in a particular patient response expectancy that becomes self-confirming, and thus influences the patient outcome (23).

During the implementation phase of the short-term MBT program at Stolpegaard Psychotherapy Centre, treatment duration seemed to influence the trial therapists’ experiences of the treatment, as highlighted in staff meetings and clinical vitiation meetings. Therefore, the purpose of this study was to examine the therapist perspective related to changing from a long-term to short-term MBT program for the treatment of outpatients with BPD, in order to gain a deeper understanding of the factors that influence the therapists’ experiences with short-term MBT.

To our knowledge, this is the first empirical study assessing the therapist perspective not only on short-term MBT but also on short-term psychotherapy for patients with BPD more generally. Thus, it appears that while most research and clinical experiences indicate that psychotherapy for BPD can be very challenging for the therapist, we do not yet have empirical evidence on the therapist experience of delivering short-term MBT. This study aims to close this empirical gap in knowledge.

### Aims

This study will provide an in-depth exploration of therapists’ experiences with short-term MBT for patients with BPD. The objectives of the present study were to investigate the following research questions:

- Do therapists experience any challenges specific to short-term MBT?
- Do therapists expect different treatment effects of short-term compared to long-term MBT?
- How can short-term MBT be improved, according to therapists?

## Methods

This study is reported according to the Standards for Reporting Qualitative Research (SRQR) guideline (24).

### Design

Semi-structured qualitative interviews were conducted with MBT therapists exploring their experiences with delivering short-term MBT to outpatients with BPD. All interviews were verbatim transcribed and analysed using thematic analysis.

### Context

This study was conducted at the Outpatient Clinic for Personality Disorders at Stolpegaard Psychotherapy Centre, Mental Health Services in the Capital Region of Denmark from March to October, 2018. At the time of data collection in the present study, a 1-year pilot phase of the Short-Term MBT Project (4) had recently ended, and enrolment to the trial had commenced. During the pilot phase, all trial therapists at the clinic received training in the short-term MBT program by trial investigators as well as national and international MBT specialists. All participants had finalized 1–2 short-term MBT groups before the time of the interviews.

#### Sampling strategy

All clinicians (*n* = 7) who had been working with short-term MBT for outpatient with BPD during the pilot phase of the MBT-RCT trial were invited to participate in this study, and all of them consented to participate and provided written informed consent.

#### Participants

Participants in this study were seven psychotherapists working at the Outpatient Clinic for Personality Disorders at Stolpegaard Psychotherapy Centre. The seven therapists were experienced in both short-term and long-term MBT. Demographic information about the participants can be found in Table 1.

**Table 1:** Demographic information about the study participants.

Demographic characteristics of study participants (*n* = 7)	
Age, mean (SD), years	50,8 (14,7)
Years of work in the field, mean (SD)	18,3 (14,4)
Years of MBT experience, mean (SD)	6,7 (2,7)
Educational background	
Psychologist	4
Psychiatrist	1
Social worker	1
Physical therapist	1

#### Interventions

The long-term (14-month) version of MBT has been implemented at the outpatient clinic for the past 10 years. The short-term (20 weeks) MBT program is overall similar to the existing long-term program, but differs structurally in the following three ways: (1) The short-term program is lower in treatment intensity (both duration and exposure), (2) the same therapists provide both group and individual sessions in the short-term program (conjoined psychotherapy), such that each of the two therapists have half of the group participants in individual therapy alongside the group sessions, whereas the group therapy and individual therapy are provided by different therapists in the long-term program (combined psychotherapy), and (3) the short-term program is structured in closed groups, in which all patients start and finish the program together, whereas the long-term program is structured as slow-open groups, in which a new patient can enter a group when another finishes (4). Seven to nine patients are included in each group in both short-term and long-term MBT. The role of the therapists in the group is to facilitate a mentalizing dialogue. If patients shift to a non-mentalizing stance, then the therapists should aim to identify this shift and bring the patient back into a mentalizing stance.

#### Data collection methods, instruments, and technologies

All interviews were conducted in person. The interviewer (ASS) held a B. Sc., in Psychology and was trained in qualitative research. A predeveloped interview guide, based on the research questions stated under *Aims*, was used for all seven interviews, but as the interviews were semi-structured, additional questions asked by the interviewer varied according to what came up in the conversation with each participant. All interviews were audiorecorded. NVivo version 12 was used for coding of the themes (25).

#### Ethics statement

All the participants received thorough information about the purpose of the study and were informed that they could withdraw their consent at any time. The names of the participants have been changed in the article to protect their anonymity.

#### Qualitative data processing and analysis

To explore the therapists’ experiences and attitudes to short-term MBT, the interviews were analyzed using thematic analysis (26). This method has also recently been used in a qualitative analysis of patients’ experiences with MBT (27). The analysis was conducted based on a hermeneutic epistemological framework. We used Nvivo software for the analysis (28).

Hermeneutics can be described as a theory of interpretation, and the hermeneutic interpretative process is dynamic and non-linear. In the process of understanding a text in a hermeneutic way, comprehension of the parts and the whole can never happen without reference to the other (29). The hermeneutic aspect of this study also entails using reflexivity actively as a tool in the analysis (30). We were aware that our assumptions as researchers could influence the findings of the study. Before analyzing the material we did not know which assumptions would be relevant. Therefore, we strove to stay reflexive and be aware of the effects of any potential preconceptions throughout the process of conducting this study (31).

The aim of thematic analysis is to identify and interpret key features of the data (32). We followed the six phases of thematic analysis proposed by Braun and Clarke (26): (1) transcribing the interview, (2) initial coding of the data, (3) searching for themes, (4) reviewing themes, (5) defining and naming themes, and (6) producing a report. In accordance with the dynamic and circular nature of the hermeneutic interpretive process, these steps were not sequential. In accordance with the hermeneutic epistemology, the analysis began at the same time as a verbatim transcription of the interviews commenced. Already at this stage, preliminary codes were formed based on notes made during transcription. Additional codes were gradually added throughout the analysis. Parallel to coding and adding new codes, condensation of the themes also began, entailing a constant “dialogue” between the themes, the transcripts and the study aims as well as critical reflection on our own expectations throughout the process. This process of coding and condensation of the themes was repeated as many times as needed until the final themes were reached, and the analysis no longer uncovered significant new material and a satisfying level of meaning saturation was reached for the purpose of answering the study aims.

#### Researcher roles, characteristics, and reflexivity

AAS participated in this study as a research assistant employed in the research unit at Stolpegaard Psychotherapy Centre but was not involved in the clinical work at the Outpatient Clinic for Personality Disorders, nor was involved in the development and implementation of short-term MBT at the clinic. Thus, the interviewer had no power or authority over the participants. SJ and SS are lead investigators of the MBT-RCT trial and were responsible for the overall design and implementation of the short-term MBT program at the clinic. SJ and SS were kept blind to the interview transcripts throughout the study. SP participated as an external academic advisor and was also blind to the interview transcripts.

AAS drafted the interview guide with ongoing supervision from SJ, SS, and SP. AAS performed all the interviews to ensure that the participants could talk more freely, since they did not have a personal relationship nor had been working with AAS in the pilot phase of the MBT-RCT trial. AAS verbatim transcribed all the interviews and performed the initial coding of data (phases 1 and two of thematic analysis as described above). Searching for themes, reviewing themes, defining, and naming themes (phases 3–5) were done in a collaboration between AAS, SJ, and SS on the blinded data. All researchers participated in producing the report (phase 6).

Since SJ and SS were involved in the design and implementation of short-term MBT program, they potentially had allegiance to this program, which could influence the research. However, particularly SS have also been working with the original long-term MBT program for many years. SJ and SS were mindful about how their role could influence the results throughout the data analysis phase. We all worked with our own preunderstandings and theoretical approaches through reflexive dialogue in the research group as well as a “dialogue” with the perspectives presented in the blinded interview transcripts.

## Results

The thematic analysis revealed four major themes: (1) *The longer the better*, (2) *Change processes can be intellectual or experiential*, (3) *Short-term therapy is hard work*, and (4) *Termination is more challenging in short-term therapy*.

### The longer the better

Several of the therapists express a conviction that the longer the patients’ issues have been present and/or the more fundamental and deep-rooted they are, the longer therapy is needed to create appreciable positive change for the patient. This is illustrated here by Susanne, who contends that the more widespread the patients’ problems are, the more difficult she finds it to get a good grasp of them in the short-term groups:

Some of the patients have problems so complex that it’s impossible to catch up with in such short-term groups.

Another therapist, Tina, also describes an experience with a short-term group where the allotted time ended up feeling too short for the subgroup of patients with more long-lasting problems:

Maybe it’s something you’ve done for a long time, and then it can be hard to do something else in just five months.

Karen demonstrates how the belief that long-lasting problems require longer-lasting therapy informed her initial resistance to short-term therapy:

I was affected by my own ideas of “okay, when they’ve had these issues for so many years, then how are we supposed to change them in five months” and things like that.

Particularly, personality change is believed by the therapists to require longer treatment. Louise presents the view that short-term therapy is mainly effective for concrete, more delimited problems:

I’m thinking that short-term therapy maybe works well for more well-defined and circumscribed problems. I mean, we know that it can reduce self-harm and suicidal risk and other very concrete goals. But I don’t know how much change in personality structures short-term therapy can bring along, I mean, if it can bring along just as much as long-term therapy. […] I think some of the more fundamental issues, like attachment trauma, are going to be really difficult to change in short-term therapy. […] Like, the more underlying attachment, I think that takes a bit longer.

Several of the therapists echo the view that patients with severe attachment-related problems need long-term therapy in order to change their underlying attachment patterns. Tina’s experience is that for some patients, short-term therapy will never be enough. When asked if she had an idea of which specific patients, this would be true for, she responds:

It’s probably the ones with several severe borderline traits, maybe, and with attachment-related trauma. I think so.

There is not complete consensus on this view though, as Susanne presents the opposite opinion saying that short-term therapy can be especially beneficial for patients with insecure attachments:

The advantage is that they don’t develop this dependence, which we see in some of the long-term groups, that we have to work very deliberately to dismantle.

Only one therapist, Maria, describes herself as being more in favor of the length of the short-term therapy in a broad sense. In her experience, the patients in her short-term groups actually have appeared to change on a deeper level:

I’ve seen that we’ve actually been successful in changing some personality-related and relationally completely fundamental things […] and then I’ve just been thinking, if you can get to that point in just half the time, then there’s no reason for giving them a year.

When asked about what they would change to improve the short-term group therapy, several therapists immediately consider prolonging the duration of the treatment, as this response by Peter illustrates:

I don’t know. I think it’s hard to say. I mean, that would be something like making it longer, but I don’t think that’s a meaningful response […] That’s not my opinion at least, that it would be unambiguously better to make the therapy longer. Although, if I had to pursue that idea, I’m inclined to wonder if 15 sessions in the MBT-G part [the group therapy minus the introductory module in the beginning] is maybe a little too short?

Michael, who otherwise expresses being an avid supporter of the short-term version of MBT, joins in on this tendency:

If you look at it from a more general perspective, then I think maybe you should make it a little longer. That’s my view. I mean, not necessarily that much more. But it’s just very quickly in and out, and maybe that’s just a few months too fast. But it’s a matter of taste. […] If I had to rethink it, I think I would have said three quarters of a year or something like that […] but I don’t have a lot to base it on, other than that sense that it actually goes by really fast.

Peter – one of the therapists who suggested prolonging the short-term treatment to a duration somewhere in between the two treatment programs – subsequently reflects on why his own experience with long-term treatment seems to be more positive:

I wonder if the long-term treatment is maybe also better, because it simply increases the chances of relevant interpersonal events happening to the patient that the patient can work with in the group […] There’s also some degree of coincidence at play in relation to what happens in these groups and in the patients’ lives, and I’ve sometimes wondered, if the progress we see with some patients isn’t necessarily because they’ve been here for eight months, but is just as much a coincidence, because we just stumbled upon a problem.

His suggestion seems to be that the advantage of longer-lasting therapy may in some cases merely be due to an increased probability for relevant material appearing in the groups as a result of a prolonged timespan.

### Change processes can be intellectual or experiential

This theme concerns the therapists’ experiences with the different types of change processes they have been a part of in their short-term compared to long-term group therapies.

Several of the therapists shares a perception that the therapeutic change with short-term patients is more superficial compared to the more stabilized and embodied transformations they have witnessed with their long-term patients. Michael expands on this distinction by describing the change processes he has seen his short-term patients go through:

For many, the treatment outcome will probably be more on the verbalized level than on an experiential level. I mean, it’s going very fast and you quickly learn some words and concepts, and I think that many of them understand “oh, so now we are in really high arousal, hold on a minute, let’s see what’s happening here”. I think they quickly catch on to that, but internalizing an experienced emotion, I think that can be hard for some of them. So in a way I think it can be a bit more superficial and intellectualizing – “oh that was pseudo-mentalizing” more than something like “oh now I have experienced that it is possible to deal with these feeling many times, therefore I have a different feeling in my stomach”.

Later in the interview, Michael compares the effect of short-term therapy to that of a good seminar; useful and beneficial for some patients, but not as internalized or in-depth regarding psychological function as the effect of the long-term therapy may be. Louise provides a useful example to illustrate the point of deep vs. superficial change when describing a specific patient’s case:

She also has this experience of having this monster inside of her that is dangerous. And then I start to doubt if we’ll be able to change that; if we are giving her the opportunity to say “You can control this monster” or if we can actually go in and say “You *are* not a monster inside” […] My immediate experience is that short-term therapy can help you handle the monster; how you can deal with it when you feel it emerging, how you can avoid it, but long-term therapy – is my theory – can create the change that makes you start to doubt whether you even have a monster inside of you.

Louise also elaborates on the idea that the experiential part of the therapeutic process may be lagging behind in the short-term groups due to the more limited time for building a therapeutic alliance and working with the relationship between the therapist and the patient:

How fundamental are the things we change? Do they achieve the trust that you are there for them and that we are able to work with this and make mistakes and repair it again, and for them to be able to then forgive you? I think reparation is a big part of MBT and of course we have a little shorter time for that now.

According to Michael, not only the relationship between the therapist and patient, but also the relationships between the different patients in the group can be of significance for the change processes taking place in the therapy:

If you get really close to each other, then the conflicts are sure to arise […] so in that way the conflict material internally in the group, which can be really meaningful to work with, has maybe been less pronounced [in the short term groups].

Louise describes a case where the two therapists in a short-term group decided to prolong one patient’s treatment in order to cement the change in his thinking, which had happened during the course of the therapy, but which they still deemed fragile at the time of termination of the initial treatment. In relation to this practice, Tina advocates for the possibility of prolonging therapy in some cases, and asserts that while concrete changes may happen in the short-term treatment, the stabilization, which she thinks only long-term treatment provides, can be more beneficial in the long run:

For some patients it just takes longer, and maybe they will get something out of it in six months – but staying in the group and having it stabilized and feeling that they’re actually working with it and being in it in another way, before we terminate? I think that means something to them later on.

This can be seen as an expression of the attitude that therapy should create change on the more experiential level, since stabilization or consolidation are mentioned as necessary requirements for a proper and justifiable termination.

### Short-term therapy is hard work

Regardless of their opinions on short-term therapy in terms of treatment effect, all the therapists seem to agree that short-term therapy at least to some extent demands more of them as therapists than the long-term therapy. One thing that differs among them is whether they perceive this to be a mostly positive or negative thing.

Susanne believes the beginning of the group therapy, when building the therapeutic relationship as well as a fruitful group culture and coherence, is more burdensome in short-term therapy. Louise presents a view of a somewhat inflexible process that extends all the way through the short-term therapy:

For me, the short-term groups are actually more challenging, because it’s more demanding when it comes to the preparation for the groups and being more focused on the goals we are working towards. In the short-term groups, it’s not really possible to revise the goals in the same way. So, if it comes to our attention after 10 sessions that we need to work with another issue, then there isn’t much time left to work with that. So that whole thing of being more focused, that’s a new way for me to work.

The therapists also describe how the increased flow of patients, made possible by short-term groups, can be a challenge, especially because of the emotionally draining nature of relational work. This makes the increased number of patients that each therapist is responsible for a challenging feat. This point is exemplified here by Louise:

This work is very strenuous on an emotional level, and it affects me that I now have to become attached to and relate to more patients than if I had long-term groups. […] I think it’s harder to become attached to the patient, and even just remember the patient, and get to that place where you have a relationship, because you don’t have the same time. I find it hard to get attached to the patients that fast.

Tina elaborates on what exactly constitutes the added workload:

I think it’s challenging to start up with four new patients at the same time. It takes a lot to establish that alliance and security and a good relationship. The practicality of writing four case formulations, and being well-prepared while establishing a mentalizing culture from the beginning, and being very focused on what it is we’re doing.

When discussing the challenges of becoming a short-term therapist, Peter contemplates if the extra time pressure, he and many other therapists experience in the short-term groups, may possibly have a positive element to it:

My experience is that it’s more straining to have a short-term group, partly because of the time element, which somehow brings about a feeling of being more in a hurry and having to be more focused – and maybe that’s a good thing, I suppose. […] If I think about it, the first time I have a long-term patient compared to a short-term patient, I think, figuratively, that I can lean back more when sitting with a long-term patient. I think I feel compelled to be a little more active with the short-term patient. And I don’t know which of the two is best. But it feels more like a pressure and more stressful, and it can be a bit heavy. On the other hand, maybe it can be an advantage because you hold yourself to it and you get stuff done.

Michael puts it more concisely:

You don’t just sit there and lean back and wait for things to happen on their own like there perhaps is a tendency to do in the longer therapies […] I think I have been more active as a therapist in the short-term groups. Time is sparse, and I’ll be damned if we don’t get something out of this, you know.

Together, Peter and Michael’s points seems to be that a therapist who is pushed slightly by having to work within a shorter time frame may become more focused and goal-oriented, which could ultimately make for better therapists. Perhaps an added sense of accountability follows, when a predetermined date of termination lies within a more foreseeable future. This may be beneficial, providing it does not result in an insecure or stressful working environment for the therapist.

### Termination is more challenging in short-term therapy

With shorter treatment durations comes an added number of groups and as a result more endings for the therapist. It is therefore perhaps not surprising that termination emerges as one of the major themes in the therapists’ accounts of their experiences with this new treatment form. Most of the therapists, when asked if they experience any specific challenges related to conducting short-term therapy, bring up difficulties regarding termination. While the majority recognize that termination can be hard in all types of therapy, most of them stress that termination feels particularly challenging when it comes to short-term therapy. For Karen, termination is largely the same process in both short-term and long-term groups, but it perhaps takes up more space in the short-term groups. She adds:

Well, you can say that the termination phase approaches faster. You know, if it’s five months of treatment, sometimes even after four months, they already start to think that it’s about to end. But the process is kind of the same. It’s the same themes. […] It’s the same things we have to go through about looking back, or the difficulties with saying goodbye and the insecurity of having to stand on your own two feet.

Peter finds termination to be more difficult in short-term groups and in relation to this highlights the emotional challenges related to termination:

It’s evident that termination in short-term therapy *is* more challenging. And again, it’s hard to say how much is our own, and how much is the patient’s, but emotionally there is more at stake by ending therapy after five months.

The therapists have found different ways to deal with the struggles with terminating therapy especially present in the short-term program. Some therapists, like Susanne, have appreciated the possibility in the pilot phase of the trial of being able to prolong the therapy, effectively transforming it from short-term to long-term therapy and thereby postponing the most difficult of the endings. Maria stuck to the amount of sessions prescribed by the short-term therapy program, but instead spread out the three follow-up sessions over a longer period of time to be able to follow the patients for longer, adding:

I would find it hard if I didn’t have these follow-up sessions. Then I would think it’s very abrupt. Yeah, and I wonder what that’s about?

After the RCT has commenced, preventing the therapists from prolonging treatment, others, like Karen, have used the practice of referring the patients to another treatment for comorbid disorders to avoid the discomfort of feeling like she is leaving the patients on their own. The therapists who make use of these strategies all describe a feeling of safety in knowing that they have done all they can and that the patient will not be left completely alone, feeling forsaken by the therapist. Michael describes how he has also struggled with terminating short-term therapy in a satisfactory way, but has found it important to stick to the principle of ending it when it’s time to end:

The challenge is also to conclude the therapy with the patients in a way where they actually feel done, right. […] So, in a way it says something about an experience for the short-term patients of having some remaining issues, which are making them push for extension. You have to be more on top of things as a therapist and say “this is the end” than you do in the long-term groups […] and then of course you have to be able to resist that pressure with your own emotions and rationality and look at what the cause of it is.

This principle might be especially imperative in cases where the patient has issues with dependency, as Maria points out:

It’s possible that we have some patients who almost become a little dependent on this place, that it becomes the only place they have […] where they forget to take part in the world outside, but instead just go from therapy to therapy. So that’s a risk with long-term therapy; creating that dependence. You don’t do that in the same way with the short-term groups, because it’s so clear to them when to stop.

Tina shares this understanding with Maria and remembers a patient of hers, who expressed that short-term therapy was the right choice for her because of her dependent traits and struggles with dependency in the past:

She said that she was very worried about the duration, when she started, and in the end she said “I think it’s good that it wasn’t longer” because then she would have become too attached to the treatment, and then stopping would have been too hard.

In line with this, Peter points out that patients’ negative reactions to termination may not necessarily require any action from the therapist, since these reactions are often rooted in the patient’s underlying dependent personality structures or may, alternatively, merely be a natural reaction to something positive ending:

I think it speaks to some sort of dependency that, you know: “Someone has begun to help me and listen to me, and it’s been so nice, so it’s hard that no one is going to be doing that anymore”. That is very understandable, but not necessarily an argument for more therapy, in my opinion.

The habit of prolonging the therapy for patients in short-term groups (assigning them to another short-term group after termination or letting them transfer to a long-term group) during the pilot phase of the RCT might be indicative of the therapists’ resistance to termination and perhaps to short-term therapy as well. While a few of the therapists have appreciated having the opportunity to do so, looking back, most feel that this practice has actually been less than beneficial for the patients, as Tina describes:

I think one out of the five times has been a good idea, in retrospect. For the remaining three to four it was, like, a very concrete solution to something that was hard for both the patients and the therapists to bear: Ending the therapy […] you couldn’t bear being in that feeling with the patient, or you think that you’re meeting them where they’re at, but it wasn’t actually what they needed after all.

In a candid remark, Peter also reflects on what is likely to motivate therapists to prolong therapy in some cases:

It’s not that we can necessarily say that they’ve improved more after a year – or at least not just because it’s a year – but because we can say with greater peace of mind: “Now we have tried for a whole year, and we have come as far as we can”. So, it’s not necessarily because they have progressed more, but because you yourself can end it feeling calmer. Because I think the termination is a lot about what we as therapists think is hard. I think this makes it easier for us.

However, the theme of termination is not only present in short-term therapy, but in long-term groups as well. Louise brings nuance to the matter by suggesting that the separation anxiety experienced by some patients might rather be related to the patients’ symptomatology and have less to do with therapy duration:

Some of the really ill patients I’ve seen can be in groups that last a year and a half, and be desperate already in the beginning, thinking about the therapy ending.

Nevertheless, Louise adds that she feels more “done” when terminating therapy with patients at the end of the long-term compared to the short-term groups.

As presented under the theme *Short-term therapy is hard work*, the therapists seem to consider short-term therapy more difficult than long-term therapy. Based on the findings from this theme it seems that issues related to termination might be a factor in this. However, rather than termination difficulties, Karen highlights the effect of having to embark on new therapeutic relationships more often than the therapists are used to:

You don’t get many weeks before it’s time to start terminating the therapy, and that can be rough. But I almost feel like hellos are harder than goodbyes in some way. I think building a good alliance, a good relationship requires a lot.

With this comment, she seems to suggest that while she experiences short-term therapy as harder for her as a therapist, this may be an effect of the added number of beginnings, where the relationship needs to be established, and less due to the issue of termination. Karen also highlights a possible positive effect of a nearer, fixed termination date that feels more imminent than in long-term therapy:

You can’t know if that thing of “it’s ending now, I should probably get to it” if that can be a motivator. I don’t know.

While most therapists agree that the termination theme was more prevalent in short-term therapy, a few of them also point out that there are challenges in relation to all endings of psychotherapy regardless of the duration, and one could speculate if the same attitudes would occur even if the termination date was pushed a few months.

When discussing the theme of termination, the topic of how much therapy is enough becomes pertinent. In order to know when it is acceptable to end the therapy, the therapists must be clear on what the therapy goals were, and if these have been satisfactorily met. The therapists have somewhat differing opinions on when therapy is terminable. Some seem to carry the conviction that the patients should preferably be completely ready to end, satisfied with the therapy, or perhaps even free of their symptoms, while others believe in an ongoing therapeutic development continuing after the therapy itself is over. Peter unfolds his own uncertainties with the issue of when it is reasonable to terminate therapy:

I can almost hear a patient saying: “Sure, I’ve gotten better, but there is still all this stuff I’m dealing with”. Something, which is hard when it comes to termination, is that it can become a bit vague and unclear where exactly we are supposed to bring them to. For example, is it a success criterion – and I think it is – that they self-harm less? But we have some patients who become less self-harming, but are still quite dysregulated emotionally, now they just have better ways to handle it, and it happens a little less, but is that the point we need to get to, or do we need to get even further?

The question of therapeutic sufficiency is connected to the theme *Change processes can be intellectual or experiential*, because it deals with the issue of which type of psychological or behavioural change is needed in order to consider the patients mentally well enough to terminate therapy. Is intellectual change sufficient – maybe because you expect that further progress will happen after the end of therapy – or should the change be experiential and stabilised for termination to be warranted? Some therapists state that they experience a greater stabilization of the therapeutic progress in long-term therapy, not to mention a greater acceptance of the termination by the patients, which they often do not see in short-term therapy. This is exemplified by the following statement by Tina:

It think that it generally means a lot to them in the long run that they don’t feel like they are being turned away too soon, but that they feel escorted somehow, and that these new things land in them, so they feel like “Okay, now, this feels fine”, and are sort of satiated.

Peter expresses some doubts as to whether the assumption of continued progress post-therapy is well-founded enough to defend a perhaps early termination:

We say to the short-term patients: “Here you have the opportunity to train your mentalization, and the idea is that if you get better at this and start working with it and become aware of what it means to mentalize and that it is something you have to practice, then you can always get better at it after you are no longer in therapy.” […] I’ve heard people say that in the context of many other therapies as well, and on one hand it makes a lot of sense. But I also think: How well do we really know that?

Maria presents the view that the difference in termination-related challenges in the two groups may be driven not only by the difference in duration, but also by the format of the therapy. She suggests that patients in the so-called slow-open groups may benefit from the way the group is structured, which makes it easier to terminate therapy, and she compares the effect to a life maturation process:

A sort of natural maturation happens […] If you start out as the new person in the group and at some point end up being the old one in the group, and new people join and such, and all your buddies have left … I mean it’s almost like a life maturation, you know, when you grow old and all your friends die and you sit there with people 30 years younger than you in a completely different place in life, then maybe you become more ready to leave.

It thus seems that the prerequisite of termination is not only getting to a place where the patients are “fully treated” or void of all symptoms of mental illness. Rather, several other different variables, such as the type of patient, the type of group and the prospective of further development after therapy can make both the therapists and the patients feel more ready for the therapy to end.

### Summary of the findings

**Figure 1:**
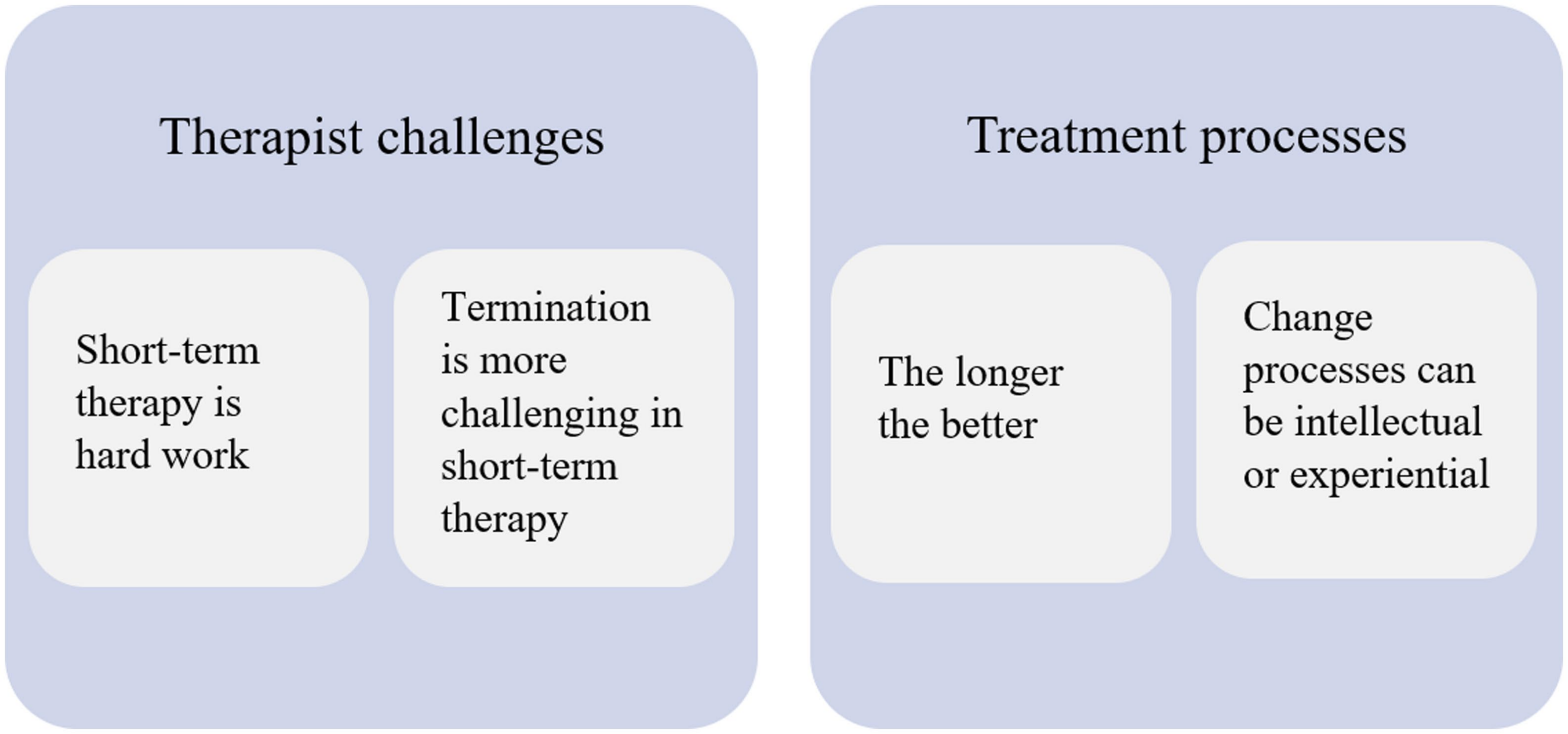
Therapist attitudes to short-term MBT illustrated by four themes and their relation to two overarching themes.

The therapists reveal that their preference for long-term therapy may both concern the well-being of the patients as well as their own work life, the emotional challenges related to being a therapist and perhaps, in some cases, countertransference reactions. To illustrate this, the four themes can be divided into two overarching considerations: (1) The therapists’ ideas of treatment processes and effectiveness for the patients, and (2) The therapists’ personal and professional challenges (Figure 1).

## Discussion

In the present study, we interviewed seven MBT therapists using semi-structured interviews about their experience with delivering short-term MBT for outpatients with BPD and about their experiences of changing from long-term to short-term MBT. Seven therapists provided informed consent and participated in this study. The interviews were verbatim transcribed and analysed using thematic analysis. The results suggest that therapists seem to have some reservations toward the short-term MBT program, which are expressed in differing ways. The following four major themes from the therapists’ experiences with short-term MBT were found in the qualitative analysis: (1) *The longer the better*, (2) *Change processes can be intellectual or experiential*, (3) *Short-term therapy is hard work*, and (4) *Termination is more challenging in short-term MBT*. Some therapists also pointed to potential advantages of delivering short-term MBT. While highlighting that short-term MBT is hard work, some also mentioned a tendency of becoming more structured and deliberate in their psychotherapy practice.

As presented in the theme ***The longer the better***, the idea that chronic and/or more severe mental disorders such as personality disorders require long-term therapy is prevalent among the therapists. The therapists’ arguments for the superiority of long-term psychotherapy often include the anxious and ambivalent quality of the patients’ attachment patterns as well as the severity of their psychiatric symptoms. However, a study by Arnevik et al. (33) suggests that the patients with the most severe disorders are not necessarily the ones benefitting from higher treatment doses, as they find no additional benefit of the more extensive treatment in terms of change at 18 months’ follow-up. However, in a subsample analysis of patients with BPD from the same study, Antonsen et al. (34) found that this group of patients benefitted more from higher treatment doses at the 6-year follow-up. Yet, one should be careful with drawing conclusions based on subgroup analyses, which should be seen as observational by nature and thus mainly hypothesis generating (35). Whether patients with more severe personality pathology (or psychopathology in general) need longer treatment is still unknown (36). A randomized clinical trial assessing the effects of six versus 12 months of dialectical behavioral therapy (DBT) for BPD patients was recently published (37) in which it was concluded, that short-term DBT was non-inferior to long-term DBT when assessing the primary outcome, total self-harm, as well as other secondary outcomes. Hence, whether this could be true for MBT as well is still unknown.

In the interviews, the therapists express their views on which change processes are most beneficial for the patients. As presented in the theme ***Change processes can be intellectual or experiential***, the general consensus among the therapists seem to be that experiential (as opposed to intellectual) change is preferable, while some therapists are also quite transparent about their own uncertainty about mechanisms of change and optimal treatment goals. The convictions held by the therapists appear reminiscent of the psychoanalytic roots of MBT. Psychoanalysis is known as a quite time-consuming type of psychotherapy compared to most other modern psychotherapies, with durations of several years of high-intensity therapy. Freud’s belief was that attempts at mere symptom extinction was far too superficial (38, 39). Instead, he likened analysis to surgery seeking to remove the problem rather than merely covering it up and posited that treatment is not finished until all obscurities are cleared up, the gaps in the patient’s memory filled in, and the precipitating causes of the repressions discovered (40). In MBT the goal of the treatment is another than that of Freud’s. The group is described as a training ground for interpersonal mentalizing with a focus on common problem areas (6). The descriptions of experiential change by some therapists in the interviews seem to correspond quite well with the term “training ground.”

Recent MBT literature presents a greater focus on epistemic trust in contrast to the pronounced focus on the attachment system in the understanding of mechanisms of change in previous times (41, 42). Luyten et al. (12) links epistemic trust to treatment duration by suggesting that patients with less epistemic trust require longer treatment in order to first stimulate trust and openness. Similarly, Bach and Simonsen (43) link lack of epistemic trust to higher severity of PD. However, the therapists in this study do not appear to use epistemic trust as a rationale for their preference for experiential change or longer treatment as their focus is more on the quality of the patients’ attachment as well as symptom severity. While attachment and epistemic trust are closely related concepts, there are important differences between placing primary importance on the emotional aspect or the learning component as a therapist (42). The therapists’ focus on attachment patterns and emotional processes, and what seems to be a perception of their role as having to embody a corrective emotional experience can perhaps explain why they find short-term MBT more difficult.

Another major part of the therapists’ preference for long-term MBT seems to stem from the experience that short-term MBT is more challenging, as evident in the theme ***Short-term therapy is hard work***. Here, it is important to add, that the therapists’ replies in the interviews are also shaped by the questions asked by the interviewer. One of the last questions in the interview invited for a discussion of the challenges in the therapists’ work life. All the therapists had, however, touched upon the topic before the question was raised, indicating that the theme is highly significant to the therapists.

Even therapists, who on the manifest level indicate that they believe short-term MBT has the potential to be as effective as long-term MBT, still express finding their role in short-term MBT more difficult. Perhaps a part of the explanation for why switching from long-term to short-term MBT seems so challenging for the therapists can be found in the therapist stance. Fonagy et al. (44) describe the MBT group leader as an authoritative and overtly mentalizing participant of the group, implying an active and processual stance. Bateman and Fonagy (6) describe the group therapist’s role as a hybrid between a floor manager and a dinner party host. In discordance with this idea of the therapist as a process facilitator, the therapists in the present study seem to personalize their role to a higher degree than prescribed by MBT literature (45, 46). Perhaps the high degree of personal involvement apparent in the interviews contributes to the therapists experiencing their role as rather emotional draining. While this may be the case for both the short-term and the long-term MBT programs, one could speculate that the therapists’ involvement with short-term patients is greater because their faith in the effect of this treatment is lower, causing them to attempt to compensate for a treatment frame which they find inadequate.

Perhaps some of the therapists’ struggles can also, at least partially, be attributed to the fact that the interviews were conducted during the implementation phase of The Short-Term MBT Project (4). The therapists in the study were accustomed to working with long-term MBT and some of them had done so for several years. It is possible that the therapists were struggling to find their footing when adapting to a shorter treatment frame. One could also consider if working with the same therapy structure for a long time may cause some therapists to become more governed by routines and perhaps more passive. One could speculate that the therapists would be less reluctant towards short-term MBT, if they had been practicing it for a longer period of time before this study. On the positive side, it is possible that the mere novelty of short-term MBT has the effect of activating the therapists in a new way. The challenge of changing habits may result in the therapists reflecting more on their role and being more deliberate in their practice, which is known to benefit patients (47). This could be part of an explanation for some of the positive effects of the new short-term MBT as highlighted by the therapists.

It appears that another part of the explanation for the experience of short-term groups as difficult can be found in the added number of terminations for the individual therapist. Terminating psychotherapy is a notoriously demanding part of practicing psychotherapy, as termination is a challenging process of individuation and separation, which involves a multitude of ethical and therapeutic issues (48–50). This is evident in the theme ***Termination is more challenging in short-term therapy***. The short-term groups have resulted in a higher turnover of patients and, accordingly, a higher frequency of terminations, which perhaps contributes to a sense that termination is a more prevalent focus in short-term therapy and possibly result in a more emotionally draining work life for the therapists. According to Bateman and Fonagy (6), one of the goals of the final phase in MBT is to increase responsibility and independent functioning. The final phase in the original 18-month MBT does, however, begin when there is still 6 months of treatment left (6). The directions presented in the MBT manual are therefore not immediately transferable, since the time available for working through termination-related challenges in this original model is far from comparable to short-term MBT.

Juul et al. (51) describe a mentalization-based approach to the challenges of terminating a therapeutic relationship with patients suffering from BPD and propose that termination challenges can often be partially attributed to therapists’ own conflicts associated with ending the therapeutic relationship. Different emotional responses and countertransference reactions such as feelings of helplessness or overinvolvement are likely to become activated when facing termination. In the termination phase, a patient may react in a psychic equivalent mode of functioning, which entails thoughts being experienced as real to the point where the patient sees it as truth, making it difficult for the patient to entertain alternative perspectives (52). This may result in feelings of abandonment which can in turn evoke feelings of guilt or helplessness in the therapist. If the patients also insist that they can only recover in the presence of a therapist, the therapist may feel overprotective and act on this feeling by deciding to prolong the therapy or refer the patients to another treatment modality, as many of the therapists in this study report having done. Therapists may also experience countertransference reactions that interfere with their ability to recognize the patients’ resources (53). With a lack of epistemic trust in the patients’ ability to reach a certain level of autonomy (45, 54), the therapist may run the risk of maintaining the patient’s belief that more therapy will always be needed and available. In these cases, the challenging endings may merely become postponed instead of mentalized (51).

As mentioned by one therapist, the emotional strain caused by a higher turnover at the clinic may also be due to a higher number of beginnings as well. Empathically relating to new patients and building epistemic trust and a working alliance can be challenging (45), perhaps especially if the therapist quickly gets intensely involved in the patient. These challenges are even more sparsely described than termination challenges in the psychotherapy literature, but they can nonetheless be of great importance to gain an understanding of going forward.

The question of when termination is appropriate is inextricably linked to the question of change processes discussed above, that is, what kinds of change the therapy should bring about and hereby what the goal of psychotherapy is. Firstly, it should be noted that the groups in the study are terminated in accordance with the assigned treatment duration. The fixed termination dates of MBT in the public health care system is in contrast to a more flexible, individual assessment, focusing on whether the relevant goals of therapy have been met. Perhaps psychotherapy with a fixed termination date calls for more flexible therapeutic goals, whereas more fixed therapeutic goals call for a flexible termination date or the option of prolonging treatment.

This study has several strengths. First, it has a high degree of external validity as it included experienced MBT therapists in an outpatient clinic for personality disorders. Second, it has a clear clinical objective, as it is based on experiences from a clinical setting and includes only therapists with direct experience with the relevant therapies as participants. Therefore, the results of the present study can be generalized to other MBT clinics, who seek to implement short-term MBT as part of their treatment service for patients with BPD. The therapists’ suggestion for improvement of short-term MBT could be taken into account when implementing the program in the future. For example how to implement measures to prevent therapist burnout as a result of a high patient load and rapid turnover as a result of the short-term format.

This study also has limitations. First, the study was conducted in a specific context including very experienced therapists who had been doing long-term MBT for a long time, and were still transitioning to the short-term MBT program. Therefore, the findings of this study may not generalize to newly set-up clinical settings or to more novice therapists. Second, the therapists who participated in the study were all part of the same team of therapists at Stolpegaard Psychotherapy Centre. It is likely that the therapists have previously discussed some of the topics from the interviews in the staff group, and thus some of the opinions expressed in the interview may also have been influenced by experiences shared through discussion among the therapists. Third, the limited number of participants in this study makes an in-depth exploration of their experiences possible, but it also raises an issue about generalizability of the findings. Qualitative studies of this nature are concerned with generation of ideas and hypotheses rather than generalizability of findings (55), leaving the question of how the experiences of the therapists in this study relates to a wider population of MBT-therapists open. Fourth, two of the authors of this study are principal investigators of The Short-Term MBT Project (SJ, SS) and could thus have blind spots regarding the qualitative analysis of the findings. This is especially important in relation to our epistemological framework which is contingent on a high level of reflexivity. However, since the researchers’ experiences are seen as a precondition for interpretation rather than biases to be completely eliminated in the hermeneutic tradition, the two researchers’ close connection with the field of investigation may therefore also be regarded as a strength. Furthermore, all discussions in the research team were based on blinded transcripts of the interviews to make sure that the participants could talk more freely. Finally, the overall questions guiding our study could also have been framed as a question of implementation science. Only few studies have investigated how outcomes of MBT are affected by changes in organization and staff (56). More studies on implementation are available for Dialectical Behavior Therapy (57, 58) although investigators still highlight that further studies are needed to gain a better understanding of how evidence-based treatments for BPD are best implemented in real-world settings.

Future research should focus on the effect of therapist expectations on patient outcomes, preferably within the context of large scale, low risk of bias randomised clinical trials. Where therapists with different allegiances and sociodemographic characteristics are directly compared. However, a limitation of such a design is that therapist allegiance could change over time, perhaps as they become more familiar with the intervention. Therefore, allegiance would have to be overseen throughout the trial period.

## Conclusion

This study suggests that the introduction of short-term MBT is associated with some reservations and some degree of resistance among the therapists. The therapists seem to experience short-term MBT as more challenging in areas such as emotional investment, time pressure, and termination. Furthermore, the therapists mostly express allegiance with long-term MBT and suspect that the treatment effects of short-term and long-term MBT differ in terms of change processes and general symptom reduction, especially when it comes to severe attachment-and personality-related pathology. When asked to point at possible improvements to the short-term MBT program, the therapists often indicate a wish for the program to be longer.

With this study, we have investigated the challenges experienced by therapists when faced with a new short-term MBT program. It is our hope that future short-term MBT initiatives can be informed by the findings of this study, which can aid in smoother implementations of similar initiatives.

## Data availability statement

The raw data supporting the conclusions of this article will be made available by the authors, without undue reservation.

## Ethics statement

Ethical review and approval was not required for the study on human participants in accordance with the local legislation and institutional requirements. The patients/participants provided their written informed consent to participate in this study.

## Author contributions

AS conducted and transcribed all interviews. All codes and themes were discussed between AS, SJ, and SS. AS wrote up the manuscript draft with ongoing supervision from SJ, SP, and SS. All authors contributed to the article and approved the submitted version.

## Funding

This study was conducted as part of a randomised clinical trial, The Short-Term MBT Project, which was funded by Trygfonden A/S (grant number: 123488) and from the Mental Health Services Research Foundation, Capital Region of Denmark (grant no. N/A).

## Conflict of interest

The authors declare that the research was conducted in the absence of any commercial or financial relationships that could be construed as a potential conflict of interest.

The handling editor ML declared a past co-authorship with the author SS.

## Publisher’s note

All claims expressed in this article are solely those of the authors and do not necessarily represent those of their affiliated organizations, or those of the publisher, the editors and the reviewers. Any product that may be evaluated in this article, or claim that may be made by its manufacturer, is not guaranteed or endorsed by the publisher.
