# Supplementary material for: Mentalizing the therapist – Therapist experiences with short-term mentalization-based therapy for borderline personality disorder: A qualitative study
Source: Front Psychiatry. 2023 Mar 16;14:1088865. doi: 10.3389/fpsyt.2023.1088865 (PMC10061093; doi:10.3389/fpsyt.2023.1088865)
Supplement: Supplementary file 1 [file Data_Sheet_1.docx]

**Interviewguide** **for the study “Mentalizing the therapist – Therapist experiences with short-term mentalization-based therapy for borderline personality disorder: a qualitative study”**

*Introduction*

- Thank you for joining us.
- I work on the MBT project, and we’ve become curious as to what attitudes you as therapists have towards the length of the therapy you provide.
- You are anonymous (of course I know who you are, but you will not be identifiable in the article).
- You can always withdraw your consent (both during and after the interview).
- The interview takes about 1 hour.
- Do you have any questions before we get started?

| **Research questions** | **Interview questions** |
| --- | --- |
| - How do therapists relate to MBT short-term therapy?   Do they expect/perceive better, worse or unchanged treatment effect compared to long-term therapy?   - What can be difficult for the therapists in relation to MBT short-term therapy?   *- Eg. to terminate the treatment?*  *- Greater patient load?*  *- To have a fixed end date from the beginning?*  *- Greater turnover of patients?*  *- Theoretical starting point/point of view?*  *- Separation anxiety*  *- Counter transference*  *- Therapeutic sufficiency*   - Have the therapists' attitudes towards MBT short-term therapy changed?   If so: What do they think has caused this change?  *- Is it in connection with the research project?*  *- Through experience with short-term therapy?*  *- Through collaboration with a researcher?*  *- The management's efforts?*  *- Joint supervision of method?*   - How can short-term therapy be improved for the therapists + patients? - Are there differences or similarities between therapists' and patients' expectations of/desire for a specific length of treatment? | What do you think about short-term therapy (in general)?  Do you think/experience that the length of the MBT group course is important for the treatment yield?  Why/How?  What do you think are the biggest advantages and disadvantages of having *short-term groups*?  What do you think are the biggest advantages and disadvantages of having *long-term groups*?  What type of group (short/long) do you find most challenging to have? Why?  Has your attitude towards/perception of short-term therapy changed?  When and what do you think caused the change?  How do you think short-term therapy can be improved? - both your experience with it + the patients' benefit  How do you think patients feel about short-term therapy? |
